# Campaign awareness and oral cancer knowledge in UK resident adult Bangladeshi: a cross-sectional study

**DOI:** 10.1038/bjc.2011.317

**Published:** 2011-08-23

**Authors:** R Croucher, S S Islam, H Nunn

**Affiliations:** 1 Queen Mary University of London, Barts and The London School of Medicine and Dentistry, Institute of Dentistry, 4 Newark Street, London E1 4AT, UK; 2 Cancer Research UK, Angel Building, 407 St John Street, London EC1V 4AD, UK

**Keywords:** oral cancer knowledge; awareness; public education campaigns; Bangladeshi

## Abstract

**Background:**

This study reports awareness of the ‘Open up to Mouth Cancer’ campaign materials and oral cancer knowledge among two UK adult Bangladeshi communities, both at high risk for oral cancer.

**Methods:**

Differences in the outcomes of campaign awareness and knowledge of oral cancer risk factors and early signs were compared between campaign and comparison areas. Home-based interviews were conducted with representative samples from both areas by bilingual interviewers. Data collected included a modified 36-item Humphris Oral Cancer Knowledge Scale and socio-demographic information. The data were collected 4 weeks after the campaign completion and analysed using χ^2^-tests and binary logistic regressions.

**Results:**

The response rate was 77%. Both awareness of the campaign materials (29.99% (95% confidence interval (CI) 15.82, 46.99) *vs* 8.12% (95% CI 6.16, 10.62)) and the mean Humphris Oral Cancer Knowledge Scale scores (13.32 (95% CI 11.06, 15.57) *vs* 8.27 (95% CI 6.59, 9.94)) were higher in the campaign area. The campaign area sample was significantly more likely to be aware of the materials (odds ratio (OR)=6.03, 95% CI 3.00, 12.1).

**Conclusion:**

Superior awareness and oral cancer knowledge was identified in the community with access to the campaign materials. Further evaluation to identify long-term campaign impact is required.

Oral cancer is recognised as a public health priority, impacting on ethnic minority groups (Scully and Bedi, 2000). Oral and pharyngeal cancer is the sixth most prevalent cancer worldwide. Oral cancer has an estimated annual worldwide mortality of almost 130 000 (Parkin *et al*, 2005; Ferlay *et al*, 2010). Between 1990 and 2007 oral cancer incidence in England rose by nearly 30%. Immigration from the Indian subcontinent is considered one reason for this (National Cancer Intelligence Network, 2010). Moles *et al* (2008) reported that women of South Asian origin were significantly more likely to have oral cancer than non-South Asians.

Primary prevention to increase awareness of risk factors and the early signs and symptoms for oral cancer is proposed. The former might lead to a reduction in incidence while the latter might lead to earlier presentation and improved survival rates. In addition to older age, the primary risk factors for oral cancers are behavioural, most usually tobacco and alcohol use. The scientific basis for the prevention of oral cancer is evolving. Paan (betel quid) chewing without tobacco is now confirmed as carcinogenic in humans (Boffetta *et al*, 2008). This is of special concern for members of South Asian communities for whom chewing paan without tobacco is prevalent and might add to an already enhanced risk for oral cancer due to their use of both smoked and chewed tobacco (Croucher, Islam and Pau, 2007).

Oral cancer also has a significant impact on individual patient quality of life and substantial patient treatment costs, often due to late presentation when the disease is at an advanced stage (Scully *et al*, 2007). Schrijvers *et al* propose that survival rates could be improved by up to 30% if people present with early cancer symptoms, which can be easily observed through visual inspection of the mouth. The impacts following from late presentation are greater on the socially deprived and it is suggested that for most cancers this deprivation gap is widening (Richards, 2008).

In Great Britain, public awareness of the risk factors for and the early signs of oral cancer is reported as suboptimal (West *et al*, 2006; Eadie *et al*, 2009). Locally organised public information campaigns using leaflets have been shown to increase oral cancer awareness levels, both among the general public and health service users (Humphris, Ireland and Field, 2001; Humphris and Field, 2003; Petti and Scully, 2007). Variation in levels of oral cancer knowledge are also reported with those at lowest risk, because of their younger age or non-smoking status, being the most likely to be aware of the risk factors and early signs. Pre-existing information deficits are thus exacerbated, contributing to current health inequalities. It is also reported that any increased levels of awareness from low-intensity campaigns are of short duration.

There are large Bangladeshi communities resident in East London. The East London local authorities of Tower Hamlets and Newham are both ethnically diverse with high levels of unemployment and deprivation. Nearly 80% of the Tower Hamlets population fall into the most deprived quintile nationally (Association of Public Health Observatories, 2007). Tobacco use, either smoked or chewed, is more common in Bangladeshi adults than the general adult population and other South Asian communities. The smoking prevalence of Bangladeshi men is 40% compared with 24% of men in the general UK adult population (Sproston and Mindell, 2006), while the cotinine validated prevalence of chewing tobacco in paan in women has been estimated as 49% (95% confidence interval (CI) 42.01, 54.98%) (Croucher *et al*, 2002). In common with men from many English black and minority ethnic groups, few Bangladeshi men set quit dates with the NHS Stop Smoking Services (The Information Centre, 2011).

The high levels of tobacco use, both smoked and chewed, may predispose UK resident Bangladeshi communities to a high risk of developing oral cancer especially as there are indications that there are low levels of awareness about risk factors for oral cancer in the community. Despite this recognised knowledge deficit about oral cancer in the community as a whole, there has been little apparent consistent effort to address this.

**Campaign development**

The Tower Hamlets Bangladeshi community was purposively selected by Cancer Research UK as the target for this low-intensity intervention as it offered the opportunity to target members of a large disadvantaged community residing within a small well-defined geographic area at high risk of oral cancer. Reflecting the wider evidence base, the campaign strategy sought to influence both individual and environmental factors. The evidence base reports that communities residing in an area where a poster campaign took place would be more likely aware of the posters compared with a comparison community resident elsewhere (Etter and Laszlo, 2005) while the Wakefield, Loken and Hornik (2010) review of the use of mass media for health note the benefits of basing campaign messages on target group research in developing campaigns. Using social marketing techniques, (Grier and Bryant, 2005) bilingual (English/Bengali) leaflets and posters were developed and distributed throughout the Tower Hamlets Bangladeshi community as part of Cancer Research UK's oral cancer awareness campaign, ‘Open up to Mouth Cancer’. The materials incorporated culturally relevant images and language, and the content was aligned to the information needs of the community to promote greater involvement with the material. The use of imagery and simple language was designed to encourage understanding, including among those with low levels of literacy. In addition, environmental factors were addressed by encouraging primary health-care professionals and community workers to articulate the campaign messages. Packs of campaign materials, each containing 25 leaflets and two posters, were distributed to general medical and dental practices and pharmacies in Tower Hamlets. Additional supplies were also sent to community-based and voluntary organisations. Community members might receive the materials through either professional recommendation or personal choice.

The objective of this study was to observe differences in awareness of the campaign materials and any significant variation in knowledge of risk factors for and early signs of oral cancer in representative samples of UK resident Bangladeshi adults recruited from the two London local authorities of Tower Hamlets and Newham, using home-based interviews. Levels of campaign awareness have been compared by socio-demographic factors, making it possible to observe differences generated by the availability of campaign materials in Tower Hamlets only. It was hypothesised that there would be differences in levels of campaign awareness and superior levels of oral cancer knowledge when respondents from the two areas were compared. The key outcomes were, therefore, awareness of the campaign materials and levels of oral cancer knowledge.

## Materials and methods

Participants in this cross-sectional study were all high-risk members of the Bangladeshi community resident in either of the two London local authorities of Tower Hamlets and Newham. Inclusion criteria for participation, in addition to being Bangladeshi or British Bangladeshi and a resident of one of these two areas, were being aged at least 30 years and practising one or more of the following health-compromising behaviours: smoking tobacco, chewing tobacco, both smoking and chewing tobacco, or chewing paan (betel quid) without tobacco.

The Bangladeshi community resident in the neighbouring borough of Newham was selected for comparison because it was known that many of the community had originally resided in Tower Hamlets and shared similar tobacco-use behaviours. While the Newham community was acknowledged as smaller in number compared with Tower Hamlets, with possible resource implications for identifying and recruiting survey participants, it was considered an important methodological benefit to be able to use the same group of specialist and experienced interviewers in both areas. Selecting an alternative Bangladeshi community outside London would have prevented this.

The sample size was chosen using the following assumptions. The most current information (West *et al*, 2006) on oral cancer awareness in UK adults was scrutinised to establish levels of correct response to questions about particular risk factors and early signs and symptoms of oral cancer. Awareness of different risk factors and early signs of oral cancer in the UK was found to vary between 35–85%. Using the most pessimistic assumption about awareness in Tower Hamlets and Newham, a significance level of 0.05 and a population estimate of 26 000 aged over 30 years, it was established that a sample of 340 completed interviews, equally divided between the two areas, would be appropriate.

Recruitment into the study took place between August and September 2006. Pragmatic focussed enumeration, a process designed to reduce the cost of identifying members of a target population for which there is no sampling frame, was used to identify addresses at which members of this community lived (Sproston and Mindell, 2006). Initial addresses to visit were selected from the current electoral register using a standardised protocol to identify Bengali names (Nicoll, Bassett and Ulijaszek, 1986). For this study, interviewers were instructed also to approach the two addresses on either side of each initial address to establish the availability of possible participants. Screening questions (Bangladeshi, age and any tobacco use) were asked to identify potential participants in the study from each address. Those identified through the screening questions as meeting the inclusion criteria were then asked to take part in the second part of the interview. For an address to be included, there had to be at least one possible respondent available for, and consenting to, the interview. Interviewers were instructed to interview up to four adults meeting the inclusion criteria from an included screened address.

Data were collected during face-to-face interviews by bilingual interviewers, who were required to use the language of choice of the respondent. The core content of the interview was provided by the Humphris Oral Cancer Scale (Humphris *et al*, 1999). This validated 36-item inventory explores respondent knowledge of risk factors for oral cancer (e.g., ‘you are more likely to get mouth cancer if you are elderly’), check ups for oral cancer (e.g., ‘a check up for mouth cancer is only going to last a few minutes’), the signs of oral cancer (e.g., ‘a sign of mouth cancer is an ulcer that does not heal’) and the UK epidemiology of oral cancer (e.g., ‘in the United Kingdom about 1700 people die of mouth cancer every year’). For this study, the inventory was modified by excluding items about UK oral cancer epidemiology and amending other items to reflect the tobacco-use and areca nut behaviours of the Bangladeshi community. Demographic information, using validated questions about age, gender, level of completed education, employment status, health service use and preferred language (Sproston and Mindell, 2006), was also collected.

Data were analysed using STATA version 10 (StataCorp., College Station, TX, USA). Question responses were initially presented as frequencies, and the χ^2^-test was used to assess differences in demographic characteristics and mouth cancer knowledge by levels of awareness of any aspect of the campaign materials, measured by a respondent reporting having seen either an oral cancer leaflet or poster in the 2 weeks before interview. Individual items from the modified Humphris Oral Cancer Scale were entered individually into binary logistic regressions, controlling for covariates. The included covariates were gender, age, education, employment status, English as a preferred language, use of medical and dental care, and area of residence. Covariates achieving the significance level of *P*<0.1 were retained in the modelling (Altman, 1997).

Data collection was approved by the Queen Mary research ethics committee. Participants gave their written consent to take part in the interview.

## Results

One thousand three hundred and fifteen addresses were screened from which 400 interviews were completed. The average number of Tower Hamlets contacts for each successful interview was 2.8 compared with 3.8 in Newham. A response rate of 77% was achieved, estimated as the proportion of successful interviews completed compared with those contacted and screened as potential participants but who refused to participate or were not available for interview after three attempts to establish contact. Following further data screening, 369 completed interviews have been included in this analysis, 199 from Tower Hamlets and 170 from Newham.

The sample was equally divided between men and women, and the mean age was 47.33 years (95% CI 45.94, 48.71 years), 25.64% (95% CI 21.8, 31.88%) reported receiving no formal education and 23.25% (95% CI 18.86, 28.31%) reported that they were employed. Twenty four per cent (95% CI 19.54, 29.23%) reported chewing paan without tobacco with the remainder either smoking (24.82% (95% CI 20.28, 29.99%)) or chewing tobacco (38.22% (95% CI 32.86, 43.88%)). The remainder described themselves as both smokers and chewers. In all, 15.9% (95% CI 12.26, 20.58%) reported their preferred language as English.

In total, 69 respondents reported awareness of the campaign materials, 57 in Tower Hamlets and 12 in Newham. Respondents from Tower Hamlets were significantly more likely to report having seen either an oral cancer leaflet or poster in the 2 weeks before interview compared with Newham respondents (29.99% (95% CI 15.82, 46.99%) *vs* 8.12% (95% CI 6.16, 10.62%)). Additional analyses indicated that of the campaign materials awareness of both the poster and leaflet was significantly (*P*<0.01) more likely in Tower Hamlets, and that respondents reporting individual poster and leaflet awareness were more likely (*P*<0.005) to have an above mean modified Humphris Oral Cancer Knowledge Scale score. Mean modified Humphris Oral Cancer Knowledge Scale scores in those with compared with those without campaign awareness were 13.32 (95% CI 11.06, 15.57) *vs* 8.27 (95% CI 6.59, 9.94).

Table 1 summarises the relationship between socio-demographic variables, health-care utilisation and campaign awareness. It demonstrates that male respondents, younger respondents, those with some completed education and those preferring to use the English language were more likely to report campaign awareness, as did respondents who had accessed medical care in the last 2 weeks and who described themselves as regular dental attenders.

### Awareness of risk factors and early signs of mouth cancer

Correct percentage responses to items about the risk factors and early signs of oral cancer are presented in Table 2. Data are presented for all respondents reporting awareness of the campaign compared with those not aware of the campaign. Four of the five statements about risk factors were significantly more likely to be answered correctly by respondents aware of the campaign compared with those respondents not aware of the campaign. Only with respect to age as a risk factor for oral cancer was the difference not significant. With respect to the four statements about the early signs of oral cancer, three, relating to white and red patches in the mouth and a lump in the neck, were significantly more likely to be recognised by respondents reporting campaign awareness. There was no significant difference in the percentages of correct responses for an ulcer not healing as an early sign between respondents with campaign awareness and those without.

### Predictors of campaign awareness

Table 3 reports the odds ratios (ORs) of predictors of campaign awareness, controlling for covariates, compared with respondents not aware of the campaign. The variables entered into the analysis were area, age, gender, education, employment, English language preference, regular dental visiting and a medical visit in the last 2 weeks. In Model 1, the predictors of age (younger), gender (males) and employment status (currently employed) lost their significance in the presence of the other variables. While completion of some education, a preference for the English language and recent use of health care remained significant, the highest OR was related to area of residence (Tower Hamlets) (OR=6.09, 95% CI 3.01, 12.33). The final regression model, Model 2, confirmed that the primary predictor of campaign awareness was residence in Tower Hamlets (OR=6.03, 95% CI 3.99, 12.10). Other covariates (respondents with some completed education, a preference for using the English language and recent use of health care) also predicted campaign awareness but at lower odds than being a Tower Hamlets resident.

## Discussion

This first community study of a sample of high-risk UK resident Bangladeshi adults, using a validated outcome measure of awareness of oral cancer risk, reports clear differences between levels of campaign awareness, the predictors of that awareness and the implications for improved oral cancer knowledge, particularly recognition of risk factors for and early signs of oral cancer. A high response rate was achieved from a homogeneous sample with similar socio-demographic characteristics. It demonstrates the opportunities to successfully increase risk awareness for oral cancer in high-risk members of a deprived community using a targeted public campaign that adopted a culturally sensitive approach.

The primary outcomes of this study have been awareness of any part of the campaign materials, whether leaflet or poster, and levels of oral cancer knowledge. It may be suggested that an important contributory factor in this was the development of bilingual culturally specific campaign materials and their distribution through a wide range of community accessed organisations serving the Tower Hamlets Bangladeshi community, as has been concluded from other oral cancer awareness campaigns (Papas, Logan and Tomar, 2004).

Study limitations should be acknowledged. First, there was the lack of a baseline measure of oral cancer knowledge in the two areas. Alternative explanations for the observed variation in awareness cannot be discounted and have been explored in the analysis, but this has demonstrated that variation in the observed levels of awareness and oral cancer knowledge after the campaign were more likely reported by respondents from Tower Hamlets, after controlling for other possible covariates. The materials were the only publically available resources to include the most recently recognised oral cancer risk factor, chewing paan (betel quid) without tobacco, and recognition of this risk factor was significantly superior in respondents reporting campaign awareness. Furthermore, the campaign materials were primarily distributed through general medical and dental practices in Tower Hamlets, and in the final regression model, a recent medical and/or regular dental visit both contributed significantly to predicting awareness.

Second, this community-based trial was carried out in two geographically coterminous local authorities. It was not possible to exclude the possibility of contamination between respondents from the two areas. Unusual patterns of response, suggestive of contamination, were identified in some Newham responses and these were subsequently excluded from the analysis.

Third, only short-term changes in knowledge from a low-intensity campaign are reported here. The impact on long-term knowledge change and behavioural outcomes following a sustained campaign has not been established and awaits identification.

There are four key findings of this study. First, the results confirm that target communities will recall a poster campaign and its key messages (Etter & Laszlo, 2005). The analysis shows that being a respondent recruited from Tower Hamlets was a key predictor of campaign awareness after controlling for all other covariates. Respondents reporting campaign awareness were also found to have superior oral cancer knowledge. This finding has also been reported by Eadie *et al* (2009) as an outcome of the West of Scotland Cancer Awareness Project.

Second, overall oral cancer knowledge scores remain suboptimal compared with the general population. Following access to an oral cancer knowledge leaflet, a sample of UK adults achieved a mean score of 33.4 on the Humphris Oral Cancer Knowledge Scale (Humphris *et al*, 1999), suggesting a uniformly high level of awareness of oral cancer in that sample, compared with the mean score of 13.3 reported here by those aware of the campaign materials. It should be acknowledged that the method of leaflet distribution varied, with participants in the Humphris study being given a personal copy of the leaflet and advised of a follow-up evaluation interview, compared with the more voluntaristic approach to campaign material distribution adopted here.

Third, some individual levels of response were found to be similar to or superior to those reported in other studies. Higher proportions of those reporting campaign awareness correctly identified a red patch and a white patch as an early sign of oral cancer and alcohol as a risk factor for oral cancer than in the general adult population of Great Britain while similar proportions as in the general adult population correctly identified tobacco smoking and oral tobacco use as risk factors (West *et al*, 2006). This finding emphasises the potential for developing appropriately designed interventions with other deprived communities where knowledge of oral cancer risk factors has been identified as low (Dodd *et al*, 2008). In any revision of the materials, the observed lack of significant difference in awareness of an ulcer not healing as an early sign of oral cancer should be addressed.

Finally, the results of other studies would predict that lower levels of campaign awareness would be found in female respondents and those with lower levels of completed education. This was not found to be the case in this study. While completing some education remained as a predictor of campaign awareness, gender did not.

In conclusion, the current findings suggest that oral cancer risk factor awareness and knowledge was greater in members of the East London Bangladeshi community who had seen oral cancer campaign leaflets and posters. A key predictor of this outcome was respondents residing in Tower Hamlets, where the campaign materials were distributed. Increased levels of awareness of both the risk factors and early signs are of public health importance as they are necessary precursors of oral cancer prevention and early intervention. The need for continued efforts to improve population awareness of primary prevention and the early detection of oral cancer remains, incorporating specific culturally sensitive efforts, to address appropriately the oral cancer information needs of deprived populations and the development of increasing inequalities in the prevention and treatment of oral cancer. This future activity should be rigorously evaluated using appropriate community-based study designs.

## Figures and Tables

**Table 1:** Campaign awareness and sample characteristics

**Variable**	**Campaign awareness (*n*=69)**	**No campaign awareness (*n*=300)**	**Significance**
*Gender % (95% CI)*			
Male	30.97 (15.84, 51.67)	69.03 (48.33, 84.16)	
Female	19.18 (8.32, 38.32)	80.82 (61.68, 91.68)	0.005
Mean age (95% CI)	44.22 (41.47, 46.96)	48.44 (46.12, 50.76)	0.03
			
*Education % (95% CI)*			
No education	10.28 (5.50, 18.42)	89.72 (81.58, 94.5)	
Some education	30.46 (16.22, 49.77)	69.54 (50.23, 83.78)	0.018
			
*Employment status % (95% CI)*			
Employed	28.50 (12.13, 53.5)	71.5 (46.5, 87.87)	
Unemployed	30.54 (15.3, 51.68)	69.46 (48.32, 84.7)	0.24
Homemaker	16.19 (6.81, 33.8)	83.81 (66.2, 93.19)	
English as preferred language % (95% CI)	54.35 (22.42, 83.07)	19.69 (16.93, 77.58)	0.015
Regular dental visiting % (95% CI)	28.67 (15.5, 46.86)	11.73 (4.50, 27.29)	0.095
Medical visit (in last 2 weeks) % (95% CI)	39.16 (29.23, 50.08)	60.84 (49.92, 70.77)	0.05
			
*Area % (95% CI)*			
Tower Hamlets	28.99 (15.82, 46.99)	71.01 (52.01, 84.18)	
Newham	8.12 (6.16, 10.62)	91.88 (89.38, 93.84)	0.005

Abbreviation: CI=confidence interval.

**Table 2:** Campaign awareness and respondent recall of risk factors for and early signs of oral cancer

**Variable**	**Campaign awareness correctly answered (%)**	**No campaign awareness correctly answered (%)**	**Significance**
More likely to get oral cancer if aged >50	25.31	16.21	0.11
More likely to get oral cancer if smoking tobacco	84.8	48.82	0.01
More likely to get oral cancer if chewing tobacco with or without paan	82.05	36.86	0.005
More likely to get oral cancer if chewing paan without tobacco	33.66	19.87	0.005
More likely to get oral cancer if drinking alcohol heavily	59.17	28.89	0.005
Sign of oral cancer: a white patch in the mouth	44.44	20.61	0.005
Sign of oral cancer: an ulcer that does not want to heal	31.77	37.39	0.06
Sign of oral cancer: a red patch in the mouth	47.31	24.99	0.02
Sign of oral cancer: a lump in the neck	12.41	15.48	0.005

**Table 3:** Predictors of campaign awareness

	**Model 1**				**Model 2**			
**Variable**	**Odds ratio**	**s.e.**	**95% CI**	**Significance**	**Odds ratio**	**s.e.**	**95% CI**	**Significance**
Age	0.997	0.015	0.97, 1.03	0.86				
Gender	1.06	0.52	0.40, 2.78	0.90				
Some level of completed education	3.64	1.84	1.35, 9.79	0.01	4.05	1.86	1.65, 9.94	0.002
Current employment	0.86	0.27	0.47, 1.59	0.63				
English language preference	4.88	1.85	2.32, 10.25	0.005	5.16	1.91	2.49, 10.67	0.005
Regular dental visiting	2.73	1.24	1.12, 6.65	0.027	2.81	1.26	1.16, 6.79	0.022
Medical visit in last 2 weeks	4.11	1.45	2.06, 8.19	0.005	3.89	1.31	2.01, 7.51	0.005
Area (Tower Hamlets)	6.09	2.19	3.01, 12.33	0.005	6.03	2.14	3.00, 12.10	0.002

Abbreviation: CI=confidence interval.
